# Editorial: Advances in perinatal and neonatal clinical pharmacology

**DOI:** 10.3389/fphar.2024.1503429

**Published:** 2024-10-16

**Authors:** Christina Gade, Jon Trærup Andersen, Tina Bergmann Futtrup, Ulrik Lausten-Thomsen

**Affiliations:** ^1^ Department of Clinical Pharmacology, Copenhagen University Hospital Bispebjerg and Frederiksberg, Copenhagen, Denmark; ^2^ Department of Clinical Medicine, University of Copenhagen, Copenhagen, Denmark; ^3^ Department of Obstetrics and Gynecology, Copenhagen University Hospital Hillerød, Hillerød, Denmark; ^4^ Department of Neonatology, Copenhagen University Hospital Rigshospitalet, Copenhagen, Denmark

**Keywords:** pediatric pharmacology, pharmacokinetics, pharmacovigilance, neonatology, perinatal exposures, clinical pharmacology

This Research Topic is focused on recent advancements within the field of perinatal and neonatal clinical pharmacology. The perinatal period is a significant developmental transition for both fetus and newborn and represents a crucial stage where pharmaceutical interventions can potentially impact not only immediate but also long-term health outcomes.

Drug therapy during pregnancy and in neonatal care is particularly complex due to the dynamic physiological changes that occur in both the fetus and the newborn child as part of the maturation process (Moerk et al., 2022). The complexity also covers different routes of drug exposure that are not isolated to drug treatment intended for the child, but also includes drug treatment intended for the mother and as such transfer of drugs across the placenta or via breast milk. Accordingly, the critical need for research to establish optimal drug choice and dosing regimens during pregnancy, and in term and preterm neonates has long been recognized (Kearns et al., 2003; Van den Anker and Allegaert, 2021).

Despite repeated calls for further research, the availability of data robust enough for clinical decision making remains scarce to healthcare professionals, often leaving physicians with no choice but to prescribe drugs off-label or even off-science (Al-Turkait et al., 2020; Gade et al., 2023). This predicament introduces safety concerns as the lack of knowledge may introduce an increased risk of adverse drug reactions in neonates (Byskov et al., 2024). This highlights the fragility of these populations and emphasizing the imperative need for not only more research but also strategic research in perinatal and neonatal pharmacology.

The scope of this Research Topic was broad, which the variety of the included papers illustrates. The methodology used in the studies was diverse including a systematic review, an *in silico* modeling study, a retrospective cohort, and a Quasi-experimental cohort study.

De Guadalupe Quintana-Coronado M et al. reviews current advancements in pharmacology for managing various medical conditions during pregnancy. The study emphasizes the complexities and risks of drug use during pregnancy, particularly due to a lack of clinical trial data and safety concerns such as birth defects, prematurity, or pregnancy loss. Ho et al. presents an *in silico* model that simulates *ex vivo* placenta perfusion of nicotine to understand its transplacental transfer, including the role of organic cation transporters. The model mimics nicotine concentration dynamics in maternal and fetal compartments, validating results with previous experimental data. The use of this and similar models could serve as a valuable tool for researching the impact of drugs on maternal-fetal health and could aid in refining replacement therapies during pregnancy.

Cai et al. examines the impact of prenatal inactivated COVID-19 vaccination on maternal and neonatal outcomes through a retrospective cohort analysis. The study includes 1,926 women, of whom 42.9% were COVID-19 vaccinated. The findings suggest that while children of COVID-19 vaccinated women had a slightly lower gestational age at delivery and a higher incidence of late preterm births, there were no significant differences in other maternal or neonatal adverse drug outcomes. The study concludes that inactivated COVID-19 vaccines appear to be safe for use during pregnancy. Montealegre-Pomar et al. illustrate that there is still a need for studies on well-known drug therapies by examining the effects of theophylline on weaning oxygen-dependent low birth weight infants enrolled in the Kangaroo Mother Care Program. While the study group does not find any significant association between theophylline use and reduced oxygen dependency days, they show that exclusive breastfeeding and weight gain were strongly associated to shorter oxygen dependency. A finding that also emphasizes the importance of non-pharmacological interventions.

The studies included in this Research Topic illustrate, albeit on a small scale, that by accommodating different study methods, we can create a comprehensive framework for understanding drug effects in perinatal and neonatal pharmacology. A multifaceted approach, utilizing all available research methods, is essential to address the complexities of perinatal and neonatal pharmacology. Advanced methods such as population pharmacokinetics modeling and *in silico* modeling offer powerful tools to achieve these goals by capturing the unique characteristics of different populations and physiological states, but the validity of the results depends on method accuracy and data quality that enters the models. Results from modeling methods may not always translate well to real-life situations, especially as the underlying perinatal physiology is currently not fully elucidated. Well-known methods such as epidemiologic studies and systematic reviews, with their known advantages and limitations, remain invaluable in structuring existing data and knowledge to consolidate evidence from different databases and studies. To overcome obstacles in perinatal and neonate research, a multifaceted approach integrating a wide range of existing methods is essential, along with innovative, multidisciplinary collaboration that can facilitate the design and implementation of clinical trials.

For the healthcare providers, it is essential that relevant research and knowledge is available and organized for practical clinical use; efforts have been made to do so by creating pediatric drug monograph (Zahn et al., 2021) and establishing Children’s Drug and Therapeutics Committees (Holst et al., 2024). Dedicated centers of excellence for perinatal and pediatric pharmacology, ideally as an international collaboration, may provide a an easy access platform for validated recommendations in clinical use. In a research context, the creation of centralized databases for sharing research ideas, data, and findings among institutions, can enhance collaboration and accelerate the pace of discovery in perinatal and neonate research. Such a network may also provide valuable feedback on research protocols and methodology.

In conclusion, the Research Topic on advances in perinatal and neonatal clinical pharmacology underscores the complexity and innovation essential for further advancing the research in these often-neglected populations. The future of research in this field calls for a multidisciplinary, collaborative approach that combines state-of-the-art techniques and technologies, while also maintaining a commitment to established research methodologies. Establishing dedicated knowledge-sharing platforms for perinatal and pediatric pharmacology can help translate research into practical solutions, promoting the ultimate goal: to improving health outcomes for our youngest patients!
